# Effect of thrombolysis in a mobile stroke unit versus in hospital for patients with ischemic stroke: A protocol for systematic review and meta-analysis of randomized controlled trials

**DOI:** 10.1097/MD.0000000000023676

**Published:** 2021-01-08

**Authors:** Jieyun Chen, Xiaoying Lin, Risheng Huang, Minyuan Luo, Yali Cai, Wenxiao Zou

**Affiliations:** a Department of Radiology, Quanzhou First Hospital Affiliated to Fujian Medical University, Quanzhou, Fujian; b J. N. Medical Laboratory, Big Data Research Center, University of Electronic Science and Technology of China, Chengdu; c Department of Neurology, Chongqing Armed Corps Police Hospital, Chongqing, China.

**Keywords:** ischemic stroke, mobile stroke unit, National Institute Health Stroke Scale scores, onset to therapy, thrombolysis

## Abstract

**Introduction:**

Ischemic stroke caused by arterial occlusion is the cause of most strokes. The focus of treatment is rapid reperfusion through intravenous thrombolysis and intravascular thrombectomy. Two acute stroke management including prehospital thrombolysis and in hospital have been widely used clinically to treat ischemic stroke with satisfied efficacy. However, there is no systematic review comparing the effectiveness of these 2 therapies. The aim of this study is to compare the effect of prehospital thrombolysis versus in hospital for patients with ischemic stroke.

**Methods and analysis:**

The following electronic databases will be searched: Web of Science, PubMed, Embase, Cochrane Library, China National Knowledge Infrastructure (CNKI), China Biology Medicine disc (CBM), Wanfang Database, and Chinese Scientific Journal Database.

The randomized controlled trials of prehospital thrombolysis versus in hospital for ischemic stroke will be searched in the databases from their inception to December 2020 by 2 researchers independently. Onset to therapy (OTT) duration and National Institute Health Stroke Scale (NIHSS) scores will be assessed as the primary outcomes; safety assessment including intracerebral hemorrhage (ICH) and mortality will be assessed as the secondary outcomes. The Review Manager 5.3 will be used for meta-analysis and the evidence level will be assessed by using the method for Grading of Recommendations Assessment, Development and evaluation Continuous outcomes will be presented as the weighted mean difference or standardized mean difference with 95% confidence interval (CI), whereas dichotomous data will be expressed as relative risk with 95% CI. If heterogeneity existed (*P* < .05), the random effect model was used. Otherwise, we will use the fixed effect model for calculation.

**Ethics and dissemination:**

Ethical approval is not required because no primary data are collected. This review will be published in a peer-reviewed journal.

**PROSPERO registration number:**

CRD42020200708

## 1 Introduction

Ischemic stroke is the leading cause of death and permanent severe functional impairment. Most cases are caused by cerebral occlusion, or emboli in the heart or in the large blood vessels that supply the brain.^[1]^ Experimental studies have shown that cell death can occur in ischemic centers a few minutes after vascular occlusion, and that in the surrounding area (penumbra) of ischemic centers, cells can develop metabolic disorders, but can be saved with appropriate treatment. Currently, the only proven and approved therapy for acute stroke is thrombolytic therapy with RtPA.^[2]^ However, even in specialized stroke centers, <5% of stroke patients receive this treatment. If the treatment can be started within 4.5 hours, the effect will be better. Thus, the main problem with acute stroke treatment is the unacceptably long delay between the onset of the disease and the recovery of blood flow to brain tissue. The mobile medic unit is an ambulance loaded with conventional emergency equipment and all associated diagnostic instruments, which are essential to make a direct decision on thrombolytic therapy where a patient is found.^[3]^ Although existing C-T equipment can be used in this mobile stroke unit, it is small in size, light in weight (<650 g K), and the refrigeration system is independent. The mobile stroke unit can be used to send patients to the hospital door instead of asking them to come to the hospital, which can save precious time on the way to the hospital and in the hospital. Thrombolysis within l hour can be realized in many patients, but the effectiveness of this mobile pawn unit depends on the presence of trained professionals and where they are used.^[4]^ In addition, telemedicine is already widely used in many telestroke networks and ensures stroke treatment close to the patient's home in rural and medically underserved areas. This is particularly effective when telemedicine is integrated into a stroke unit concept. While telemedically based thrombolysis therapy has become routine practice for many years, practical implementation of comprehensive mechanical thrombectomy and the related processes remains challenging.^[5]^ The main tasks for the future further include development of a structured stroke aftercare system in neurologically underserved areas and permanent assurance of high-quality stroke care in telemedically connected sites. In rural areas it may be more effective than in large cities with a high concentration of hospitals with many 24 hours a day c-T services. Based on the concept of “time is brain,” the mobile stroke unit proposed here provides a novel solution.^[6]^ By shortening the delay between the onset of cerebral ischemia and the decision of treatment, the use of mobile stroke units can save ischemic brain tissue, thereby reducing the patient's personal suffering and lifetime prescription. The cost savings of caring for patients over years to decades would far outweigh the additional costs of using mobile stroke units in the first hours of the illness.

## 2 Methods

### 2.1 Study registration

This systematic review and meta-analysis protocol was registered in PROSPERO (CRD42020200708) at https://www.crd.york.ac.uk/PROSPERO/#myprospero

### 2.2 Inclusion criteria for study selection

#### 2.2.1 Type of study

We will estimate the research literature according to the criteria of the review objectives and participants, interventions, comparisons, outcomes (PICO). Randomized controlled trials (RCTs), comparing the effects of ambulance-based CT scanner on time and NIHSS^[7]^ versus in hospital.

#### 2.2.2 Types of participants

Patients of any sex or age or race or nationality with one or more stroke symptoms according to the modified recognition of stroke in the emergency room (ROSIER) scale.^[8]^

The exclusion criteria were shown as follows:

(1) Replicated studies.
(2) Meta-analysis and study protocols were excluded from the results.
(3) Studies included in this meta-analysis.

#### 2.2.3 Types of outcome measures

Major data: Onset-to-treatment time, NIHSS scores Safety assessment including intracerebral hemorrhage (ICH) and mortality.

### 2.3 Data sources

The main sources of information to be obtained in this study include electronic resource databases, trial registries, and retroactive references.

### 2.4 Electronics searches

The following electronic databases will be searched: Web of Science, PubMed, Embase, Cochrane Library, China National Knowledge Infrastructure (CNKI), and China Biology Medicine disc (CBM), Wanfang Database and Chinese Scientific Journal Database (VIP). Searching for other resources.

### 2.5 Search strategy

Comprehensive search strategy will be developed by combining medical subject headings and keywords “Ischemic stroke” “Cerebral stroke” “Mobile stroke” “Unit Telemedicine” and the restricted study type was randomized controlled trial, with a time limit of 2010 to present. Meta-analysis and study Protocol are excluded from the retrieved results.

### 2.6 Data collection and analysis

#### 2.6.1 Selection of studies

**Figure 1:**
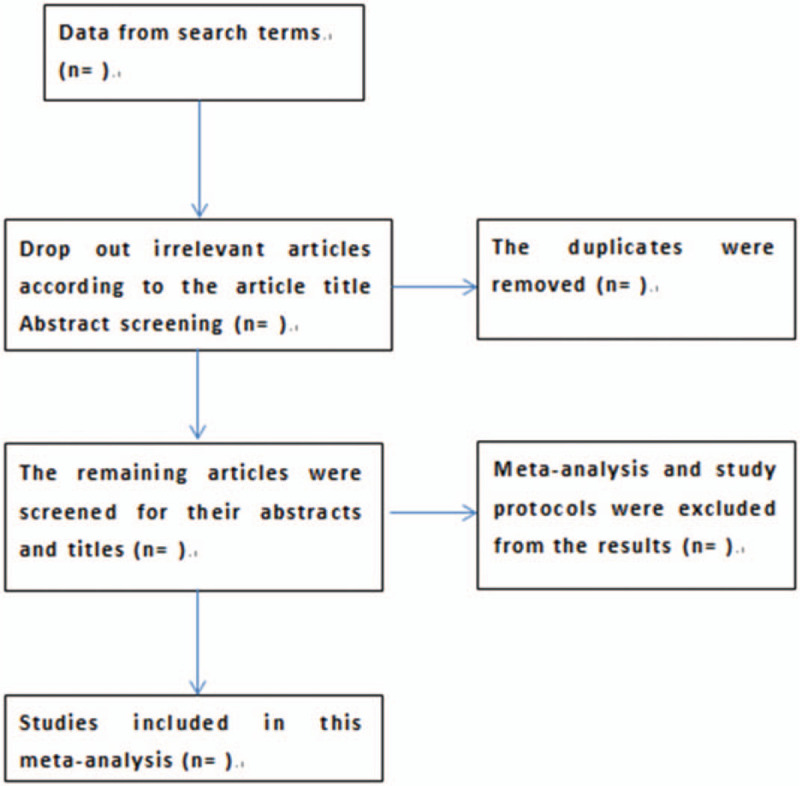
Flow diagram of studies selection.

The researchers will import the retrieved documents into the endnote database to eliminate duplicate data. The 2 reviewers selected eligible studies and independently checked against the inclusion criteria. Any differences will be resolved by consensus or consultation with a third independent researcher. The selection process is in Fig. 1.

#### 2.6.2 Data extraction and management

Two review authors will independently use a standardized form for extracting data of the included articles. The following data were extracted: general information (e.g., authors, year, and published country), details of study (e.g., study design, inclusion and exclusion criteria, blinding, randomization, and sample size), participant characteristics (e.g., age and number of subjects), and description of interventions, types of outcomes assessed, adverse events, and other detailed information. If necessary, we will contact the corresponding authors of trials for further information.

#### 2.6.3 Assessment of risk of bias and reporting of study quality

Risk of bias 2.0 will be used to evaluate the quality and risk of bias in the ultimately included studies by 2 authors independently. Review Manager (RevMan) version 5.3 will be used to present the results with a risk of bias graph and risk of bias summary.

#### 2.6.4 Dealing with missing data

We will try to contact the authors including the study to find missing or incomplete data via email. However, if missing data are not available, the study will be excluded from the analysis.

#### 2.6.5 Assessment of heterogeneity

Statistical heterogeneity will be detected by the *I*^2^ statistic and chi-squared test. *P* < .1 of the chi-squared test or *I*^2^ > 50% indicates the possibility of statistical heterogeneity among the studies.

If the included studies have existing heterogeneity, a random-effects model will be used. Otherwise, we will use a fixed-effect model for calculation

#### 2.6.6 Assessment of reporting bias

When <10 articles were included, we will use the funnel plots to assess the potential reporting biases.

#### 2.6.7 Data synthesis

Review Manager 5.3 will be employed for meta-analysis. When statistical heterogeneity is low among the results, the fixed-effects model will be used for the meta-analysis; otherwise, we will use the random-effects model.

#### 2.6.8 Subgroup analysis

If there is significant heterogeneity in the included trials, then we will conduct a subgroup analysis based on mobile stroke unit or in hospital for patients with/without additional treatment.

#### 2.6.9 Sensitivity analysis

The sensitivity analysis will be conducted to identify the review conclusions according to the following criteria: missing data, sample size, heterogeneity qualities, and statistical model.

#### 2.6.10 Grading the quality of evidence

This method will be used to assess the level of evidence for the grading of the proposed assessment. The assessment is divided into 4 levels possible ratings: very low, low, medium, or high.

### 2.7 Ethics and dissemination

Ethical approval will not be needed because no primary data are collected. Our results will provide clear evidence to evaluate the effect of ambulance-based CT scanner on time and NIHSS for patients with ischemic stroke.

## 3 Discussion

The management of ischemic stroke focuses on rapid reperfusion with intravenous thrombolysis and endovascular thrombectomy. Precious time can be saved by using the mobile stroke unit on the way to the hospital and in the hospital compared with the traditional way, which is the keypoint to treat ischemic stroke.^[6,9]^ Consequently, the comparison of the efficacy and safety will be made between ambulance-based CT scanner on time or NIHSS and in hospital for patients with ischemic stroke. This systematic review and meta-analysis will provide high-quality evidence-based medicine to evaluate the effect of ambulance-based CT scanner on time and NIHSS for patients with ischemic stroke.

## Author contributions

**Conceptualization:** Jieyun Chen, Minyuan Luo, Wenxiao Zou.

**Data curation:** Xiaoying Lin.

**Formal analysis:** Xiaoying Lin, Wenxiao Zou.

**Funding acquisition:** Jieyun Chen, Xiaoying Lin.

**Investigation:** Risheng Huang.

**Methodology:** Risheng Huang, Minyuan Luo.

**Project administration:** Jieyun Chen.

**Resources:** Jieyun Chen, Risheng Huang, Wenxiao Zou.

**Software:** Yali Cai.

**Supervision:** Yali Cai.

**Validation:** Yali Cai, Wenxiao Zou.

**Visualization:** Jieyun Chen, Yali Cai.

**Writing – original draft:** Jieyun Chen.

**Writing – review & editing:** Jieyun Chen, Minyuan Luo.

## References

[1] Prabhakaran S Ruff I Bernstein RA Acute stroke intervention: a systematic review. JAMA 2015;313:1451–62. 25871671 10.1001/jama.2015.3058

[2] Lapchak PA Development of thrombolytic therapy for stroke: a perspective. Expert Opin Investig Drugs 2002;11:1623–32. 10.1517/13543784.11.11.1623 12437508

[3] Walter S Diagnosis and treatment of patients with stroke in a mobile stroke unit versus in hospital: a randomised controlled trial. Lancet Neurol 2012;11:397–404. 22497929 10.1016/S1474-4422(12)70057-1

[4] Ebinger M Effect of the use of ambulance-based thrombolysis on time to thrombolysis in acute ischemic stroke: a randomized clinical trial. JAMA 2014;311:1622–31. 24756512 10.1001/jama.2014.2850

[5] Ebinger M Effects of golden hour thrombolysis: a Prehospital Acute Neurological Treatment and Optimization of Medical Care in Stroke (PHANTOM-S) substudy. JAMA Neurol 2015;72:25–30. 25402214 10.1001/jamaneurol.2014.3188

[6] Fassbender K Mobile stroke units for prehospital thrombolysis, triage, and beyond: benefits and challenges. Lancet Neurol 2017;16:227–37. 28229894 10.1016/S1474-4422(17)30008-X

[7] Fischer U NIHSS score and arteriographic findings in acute ischemic stroke. Stroke 2005;36:2121–5. 16151026 10.1161/01.STR.0000182099.04994.fc

[8] Nor AM The Recognition of Stroke in the Emergency Room (ROSIER) scale: development and validation of a stroke recognition instrument. Lancet Neurol 2005;4:727–34. 16239179 10.1016/S1474-4422(05)70201-5

[9] Ehntholt MS Mobile stroke units: bringing treatment to the patient. Curr Treat Options Neurol 2020;22:5. 32025945 10.1007/s11940-020-0611-0

